# Copper Functionalized SnSe Nanoflakes Enabling Nonlinear Optical Features for Ultrafast Photonics

**DOI:** 10.1002/advs.202401218

**Published:** 2024-07-24

**Authors:** Ke Ren, Hualei Yuan, Zhongben Pan, Zongsheng Li, Han Pan, Hongwei Chu, Dechun Li

**Affiliations:** ^1^ School of Information Science and Engineering, and Key Laboratory of Laser and Infrared System of Ministry of Education Shandong University Qingdao 266237 China; ^2^ Qingdao Institute of Bioenergy and Bioprocess Technology Chinese Academy of Sciences Qingdao 266101 China

**Keywords:** copper functionalized tin selenide, high‐harmonic generation, mode‐locked fiber lasers, nonlinear optical property, nonlinear pulse dynamics, saturable absorber

## Abstract

This study enhances the ultrafast photonics application of tin selenide (SnSe) nanoflakes via copper (Cu) functionalization to overcome challenges such as low conductivity and weak near‐infrared (NIR) absorption. Cu functionalization enhances concentration, induces strain, and reduces the bandgap through Sn substitution and Sn vacancy filling with Cu ions. Demonstrated by density functional theory calculations and experimental analyses, Cu‐functionalized SnSe exhibits improved NIR optical absorption and superior third‐order nonlinear optical properties. Z‐scan measurements and femtosecond transient absorption spectroscopy reveal better performance of Cu‐functionalized SnSe in terms of nonlinear optical properties and shorter carrier relaxation times compared to pristine SnSe. Furthermore, saturable absorbers based on both SnSe types, when integrated into an erbium‐doped fiber laser, show that Cu functionalization leads to a decrease in pulse duration to 798 fs and an increase in 3 dB spectral bandwidth to 3.44 nm. Additionally, it enables stable harmonic mode‐locking of bound‐state solitons. This work suggests a new direction for improving wide bandgap 2D materials by highlighting the enhanced nonlinear optical properties and potential of Cu‐functionalized SnSe in ultrafast photonics.

## Graphical abstract

The article examines enhancing tin selenide (SnSe) for ultrafast photonics through copper (Cu) functionalization. Calculations and experiments confirm the enhanced near‐infrared optical properties and nonlinear optical performance after Cu functionalization. Cu‐functionalized SnSe‐based saturable absorbers excel in erbium‐doped fiber lasers, offering shorter pulse durations and stable harmonic mode‐locking, underscoring its potential in advancing ultrafast photonics.

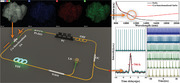

## 1 Introduction

Ultrashort pulsed lasers are in high demand in both industrial production and scientific research settings. Saturable absorbers (SAs) with ultrafast response time and low optical loss are a critical component in passively mode‐locked lasers enabling the laser to achieve highly efficient ultrashort pulses.^[^ ^1^, ^2^, ^3^, ^4^ ^]^ Up to date, one of the most successful SAs is undoubtedly the semiconductor saturable absorbers (SESAMs). However, SESAMs suffer distinct drawbacks including the limited wavelength tuning range and a complex manufacturing process. To broaden the working wavelength region, the zero‐bandgap graphene was proposed to realize mode‐locking in lasers.^[^ ^5^ ^]^ Owing to the excellent physicochemical properties and broadband optical nonlinearities, diverse innovative 2D materials have been thereafter extensively investigated in ultrafast photonics, such as black phosphorus (BP),^[^ ^6^ ^]^ MXenes,^[^ ^7^, ^8^, ^9^ ^]^ transition metal oxides (TMOs)^[^ ^10^ ^]^ and metal dichalcogenides (TMDs).^[^ ^11^ ^]^ However, these 2D materials exhibit their inherent drawbacks, including graphene's relatively lower thermal damage threshold, the poor long‐term operational stability of black phosphorus and MXenes, and the wider band gap of TMOs and TMDs, resulting in weaker optical absorption in the near‐infrared (NIR) band. Thus, the pursuit of new materials is essential to sustain exploration. Moreover, improving the performance of existing materials through modifications and adjustments remains a promising approach.

Group IV metal monochalcogenides possess an orthorhombic structure at room temperature and display asymmetric weak and strong bonding. The weak interlayer coupling can be beneficial for van der Waals structures, leading to easy exfoliation into 2D layered nanomaterials. Additionally, the strong intralayer bonding in the Group IV metals monochalcogenides leads to the noncentrosymmetric polarity and the ionic‐potential anharmonicity, inducing spontaneous in‐plane electrical polarization and ferroelectricity.^[^ ^12^, ^13^, ^14^ ^]^ As a representative member of Group IV metal monochalcogenides, 2D SnSe has drawn a lot of attention due to its non‐toxicity, abundant natural reserves of its constituent elements, and better environmental stability.^[^ ^15^ ^]^ To date, 2D SnSe with excellent thermoelectric, ferroelectric, and photoelectric properties has been widely studied in scientific applications in thermoelectricity, photodetectors,^[^ ^16^ ^]^ ultrafast photonics,^[^ ^17^ ^]^ and energy storage.^[^ ^18^ ^]^ Monolayer SnSe is composed of two atom layers with an atomic structure akin to black phosphorus but with superior oxidation resistance. Weak van der Waals forces link the layers of 2D SnSe, while strong Sn‐Se bonds exist between them, ensuring excellent monolayer stability. Moreover, 2D SnSe nanoflakes exhibit significant in‐plane structural anisotropy, which contributes to their superior polarization‐related nonlinear optical properties.^[^ ^19^ ^]^ Recent studies have revealed the broad nonlinear optical properties of 2D SnSe within the 0.8–3 µm range for ultrafast photonic applications.^[^ ^20^, ^21^, ^22^, ^23^ ^]^ However, selenium (Sn) vacancies in 2D SnSe nanoflakes result in long‐lived traps, leading to low conductivity, relatively small photocurrent, and extended recovery time.^[^ ^24^, ^25^ ^]^ Additionally, the monolayer SnSe has a band gap of ≈1.51 eV,^[^ ^23^ ^]^ which also results in weak optical absorption in the NIR region beyond 1000 nm. To address this limitation, strategies such as strain engineering and doping have been proven to be effective.^[^ ^26^ ^]^ Among various doping materials, Copper (Cu) has been found to be particularly effective in modifying the bandgap.^[^ ^27^ ^]^ Here, a Cu‐functionalized composite engineering approach has been devised to adjust the bandgap of 2D SnSe and enhance its NIR optical absorption via a dual mechanism. Initially, the inclusion of Cu fills the Sn vacancies, mitigating defect scattering that impedes carrier mobility, while introducing additional hole carriers, thereby increasing the carrier concentration.^[^ ^28^, ^29^ ^]^ This elevation in carrier concentration propels the Fermi level deeper into the valence bands of SnSe, facilitating the activation of more valence bands for electrical transport. Such interactions result in the narrowing of the bandgap, thereby optimizing the material for applications that demand superior electrical transport and optical absorption. Furthermore, the Cu functionalization process introduces strain into the SnSe crystal structure due to the smaller ionic radius of Cu compared to Sn, causing local lattice distortions and strain contrasts. This induced strain markedly influences the band structure of SnSe, further contributing to the bandgap narrowing. Theoretical analyses have demonstrated that even a minimal compressive strain, such as 1%, can significantly reduce the bandgap.^[^ ^28^ ^]^ Consequently, Cu functionalization emerges as a comprehensive strategy that refines SnSe's properties for improved utility in fields like ultrafast photonics and thermoelectric, enhancing its electrical and optical performance and underscoring a strategic method for the electronic engineering of SnSe.

In this work, Cu‐functionalized composite engineering was employed to synthesize Cu‐functionalized SnSe. Theoretically, density functional theory (DFT) calculations were performed for both pristine SnSe and Cu‐functionalized SnSe. The calculation results of the projected density of states (PDOS) showed that the bandgap of SnSe greatly narrowed after Cu‐functionalization, which is expected to produce a larger nonlinear optical response.^[^ ^30^ ^]^ Experimentally, plenty of characterization was performed to confirm the morphology, crystallinity, and optical absorption effect of Cu‐functionalized SnSe. Our systematic investigations revealed significant improvements in the ultrafast nonlinear optical responses of SnSe in the NIR band after Cu functionalization, as indicated by the shortened carrier relaxation time and enhanced nonlinear optical properties. Furthermore, SAs based on both SnSe and Cu‐functionalized SnSe were integrated into the same Erbium‐Doped Fiber Laser (EDFL), and stable conventional soliton (CS) mode‐locking operations were obtained. The output pulse duration with Cu‐functionalized SnSe SA was depressed to ≈800 fs, surpassing not only the performance of pristine SnSe (1.381 ps) in the same EDFL but also that of most saturable absorbers based on 2D materials with the same structure. Moreover, with the Cu‐functionalized SnSe SA, the EDFL could operate stably in the 53rd‐harmonic mode‐locking of bound‐state (BS) soliton operation. Our work indicates that Cu‐functionalization could be a promising approach to enhancing the optical performance of SnSe in the NIR region.

## 2 First Principle Calculation

The DFT method is widely acknowledged for its high accuracy in calculating the electronic structure of solids.^[^ ^31^, ^32^, ^33^ ^]^ In this study, the ground state of SnSe and the Cu‐functionalized SnSe structure were simulated using the Vienna Ab initio Simulation Package with projector augmented wave potentials.^[^ ^34^, ^35^ ^]^ The simulations applied the Generalized Gradient Approximation and adopted the Perdew‐Burke‐Ernzerhof (PBE) functionality for the exchange correlations.^[^ ^36^ ^]^ The structure of Cu‐functionalized SnSe was a 1 × 1 × 5 superlattice and 10% Sn was substituted by Cu. The geometry optimization involved the relaxation of the lattice constants and atomic positions, with a plane‐wave cutoff of 350 eV. The Monkhorst‐Pack method was used to generate the K‐grid of the Brillouin zone with an accuracy of 0.03 × 2π Å.^[^ ^37^ ^]^ The lattice was optimized for energy and atomic forces, with convergence criteria set to 10^−6^ eV for energy and 0.01 eV Å^−1^ for atomic forces. The frequency‐dependent complex‐dielectric function was evaluated using the random phase approximation (RPA) method, which derived the expression for the absorption coefficient by transforming the real and imaginary parts through the Kramers‐Kronig relation.^[^ ^38^, ^39^ ^]^

**Figure 1:**
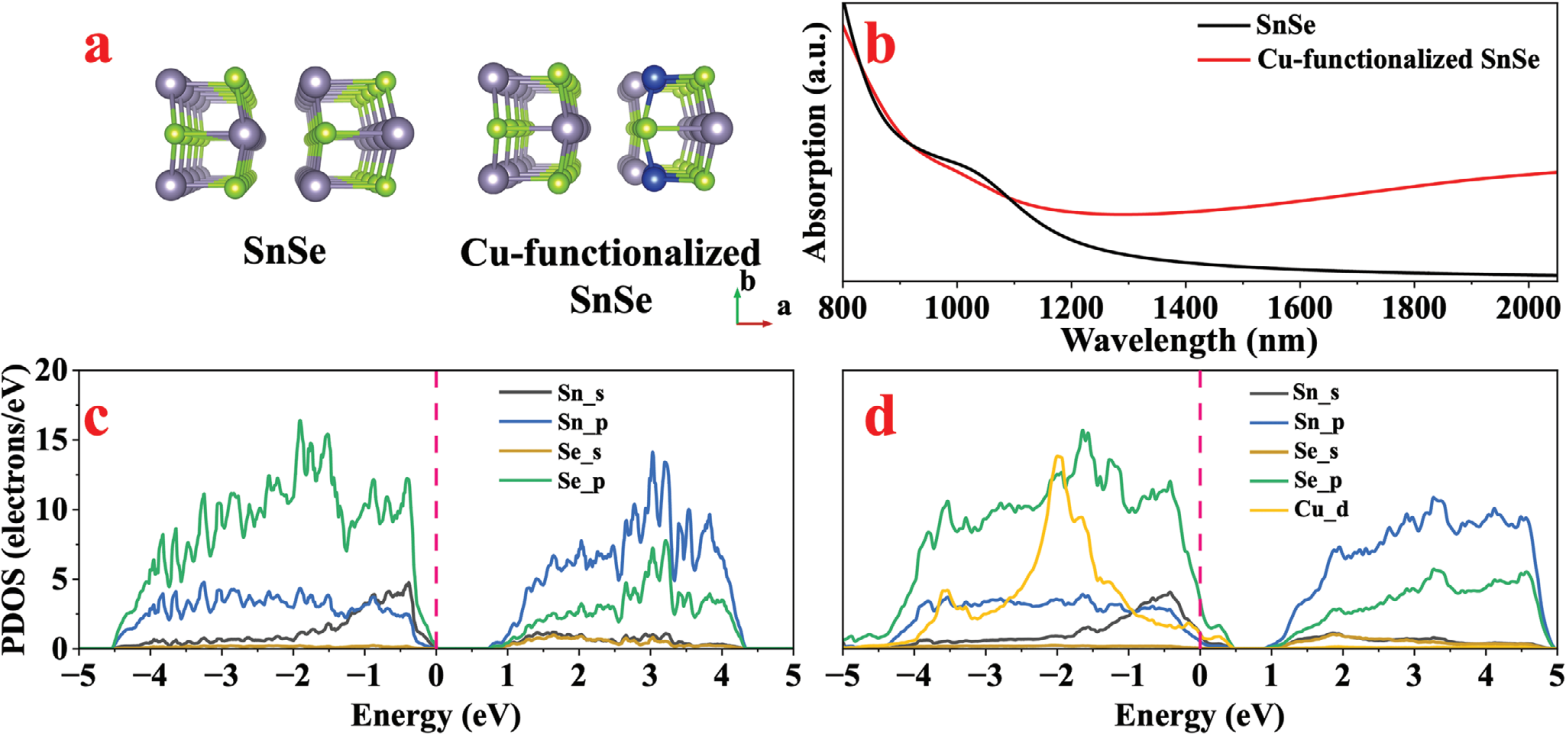
a) The structure of SnSe and Cu‐functionalized SnSe, b) The absorption spectrum of SnSe and Cu‐functionalized SnSe. Projected density of states (PDOS) plots for c) SnSe compared with d) Cu‐functionalized SnSe. The positions of the Fermi levels are labeled in the figure.

The orthorhombic crystal structure of the SnSe and Cu‐functionalized SnSe superlattices is shown in **Figure** **1**a, the optimized lattice parameters of the SnSe superlattice are *a* = 11.66 Å, *b* = 4.13 Å, and *c* = 23.5 Å, respectively and the corresponding bond length between Sn and Se is ≈2.8 Å, which are in agreement with previous literature results.^[^ ^40^, ^41^ ^]^ And the Cu─Se bond length in Cu‐functionalized SnSe is shorter than that of Sn─Se, This reduction can be attributed to the smaller atomic radius of Cu and the larger electronegativity difference between Cu and Se. Furthermore, the absorption properties of both materials are illustrated in Figure 1b, the Cu‐functionalized SnSe has a stronger optical absorption than SnSe in the NIR region, in particular, introducing Cu ions enhances the NIR absorption capacity of SnSe. To further investigate the effect of Cu‐functionalization on the electronic properties of SnSe, the calculation of the PDOS was performed for both structures, as shown in Figure 1c,d. The results indicate that the bandgap of bulk SnSe is 0.56 eV, which is lower than the experimental value (≈0.9 eV).^[^ ^42^ ^]^ This discrepancy is due to the fact that PBE typically underestimates the bandgap.^[^ ^43^ ^]^ At lower energy levels, p‐orbitals of Sn and Se form bonds; near the valence band maximum of SnSe, s‐s and s‐p orbitals of Sn and Se form antibonding states.^[^ ^44^ ^]^ Cu‐functionalization significantly affects the distribution of the valence band density of states by hybridizing with the s and p orbitals of Sn and the p orbitals of Se to form bonds while forming antibonds with the p orbitals of Se near the Fermi level. In addition, the shift of the Fermi level toward the valence band indicates metallic behavior similar to that of p‐type semiconductors. This shift introduces extra holes into the system and creates a pseudogap (≈0.4 eV), a feature recognized in potential thermoelectric materials.^[^ ^27^, ^45^, ^46^ ^]^ Narrowing the bandgap also leads to increased infrared absorption in Cu‐functionalized SnSe.

## 3 Preparation and Characterization

### 3.1 Preparation

The Cu‐functionalized SnSe microbelts were synthesized via the solvothermal method in a high‐temperature, high‐pressure autoclave using an ethylene glycol sodium hydroxide solution, Na_2_SeO_3_, SnCl_2_·2H_2_O and CuCl_2_ were used as precursors. After solvothermal synthesis, the secondary phase was successfully removed through sonic separation and centrifugation.^[^ ^47^ ^]^ To fabricate the nanoflakes, the microbelts were ground into a fine powder, and 5 mg of powder was mixed with 10 mL of ethanol in a 1:2 ratio. The ethanol solution underwent a 10‐h ultrasonic separation process at 100 W. The ice bath method was used to maintain a low temperature during the ultrasonic separation process. Following sonication, the ethanol solution underwent centrifugation at a rotational speed of 3000 rpm for 15 min, and the supernatant was extracted to obtain the nanoflakes solution without any unexfoliated bulk materials. The procedure was repeated to prepare SnSe nanoflakes.

### 3.2 Morphology Characterizations

**Figure 2:**
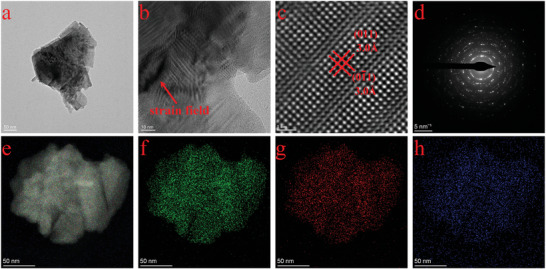
a) Low‐magnification TEM image of 2D Cu‐functionalized SnSe nanoflakes. b) High‐magnification TEM image of a 2D Cu‐functionalized SnSe nanoflakes, showing a significant strain field. Corresponding c) HRTEM image and d) SAED pattern e) STEM image of a single sample and corresponding EDS elemental mapping of f) Sn, g) Se, and h) Cu.

Systematic characterizations were conducted to analyze the microstructure and variations of Cu‐functionalized SnSe nanoflakes. The transmission electron microscopy (TEM) images in **Figure** **2**a display a well‐exfoliated 2D‐layered structure of the nanoflakes, indicating good nanoflake quality. The TEM image at higher magnification depicted in Figure 2b shows the presence of extensive strain fields, possibly induced by the size difference between Cu ions and Sn ions and the local non‐uniform distribution of Cu‐functionalization. In Figure 2c, the high‐resolution TEM (HRTEM) image displays lattice spacings of 3.0 Å for both (0 1 1) and (0 1̄ 1) crystal planes. Corresponding to Figure 2d, the selected area electron diffraction (SAED) pattern indicates that the plate has an orthorhombic structure with a (0 1 1) surface. To observe the elemental distribution of the Cu‐functionalized SnSe nanoflake sample, scanning transmission electron microscopy (STEM) with energy‐dispersive X‐ray spectroscopy (EDS) was used. Figure 2e–h shows the EDS mappings of the overlapped image of all elements, including Sn, Se, and Cu. The distribution of elements suggests effective Cu functionalization in the SnSe system and the local non‐uniformity of Cu is visible.

**Figure 3:**
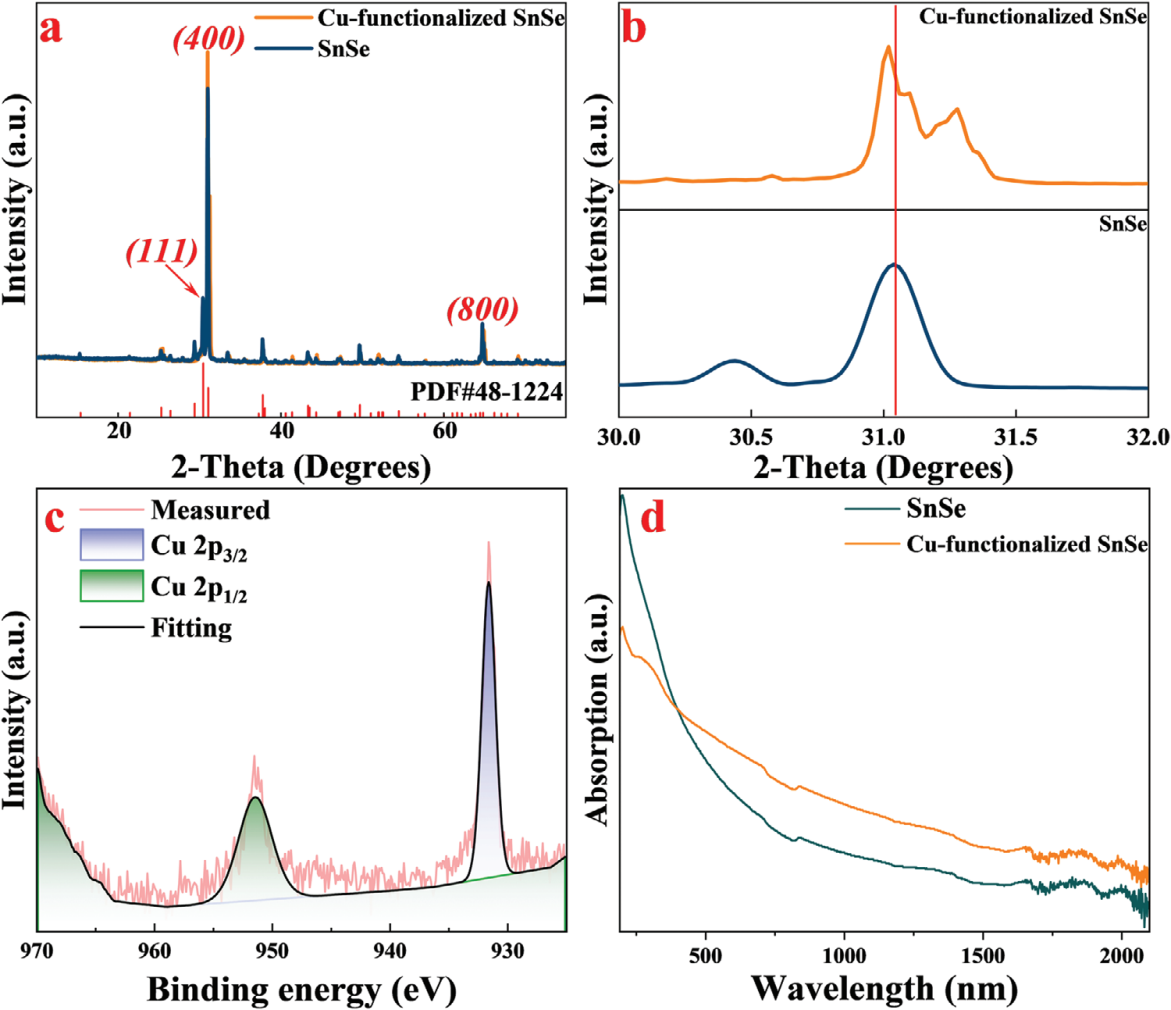
XRD patterns of a) SnSe and Cu‐functionalized SnSe Comparison. b) Magnified XRD patterns to observe the peak deviation at 400. c) High‐magnification XPS pattern of Cu 2p of the Cu‐functionalized SnSe d) optical absorption spectrum of SnSe and Cu‐functionalized SnSe from the UV to NIR band.

The X‐ray diffraction (XRD) patterns of SnSe and Cu‐functionalized SnSe powders are shown in **Figure** **3**a. Both patterns correspond exclusively to the orthorhombic‐structured SnSe, as identified by the Standard Identification Card, JCPDS 48–1224. The (111) diffraction peak weakens while the (400) diffraction peak strengthens, indicating an increased anisotropy after Cu‐functionalization.^[^ ^48^ ^]^ Figure 3b shows the (400) peaks shift toward a higher degree after Cu‐functionalization, indicating that Cu atoms merge into the SnSe lattice,^[^ ^47^ ^]^ which is caused by Cu^2+^ substituting the position of Sn^2+^, which induces a decrease in lattice parameters. Figure 3c shows two distinct peaks corresponding to Cu 2p^1/2^ and Cu 2p^3/2^, indicating successful Cu‐functionalization in SnSe. Figure 3d demonstrates that the Cu‐functionalized SnSe nanoflakes exhibit stronger absorption in the NIR region than the SnSe nanoflakes. This result is consistent with the experiment conducted by Ye et al.^[^ ^49^ ^]^ and our DFT calculation result.

### 3.3 Transient Absorption Characteristic

**Figure 4:**
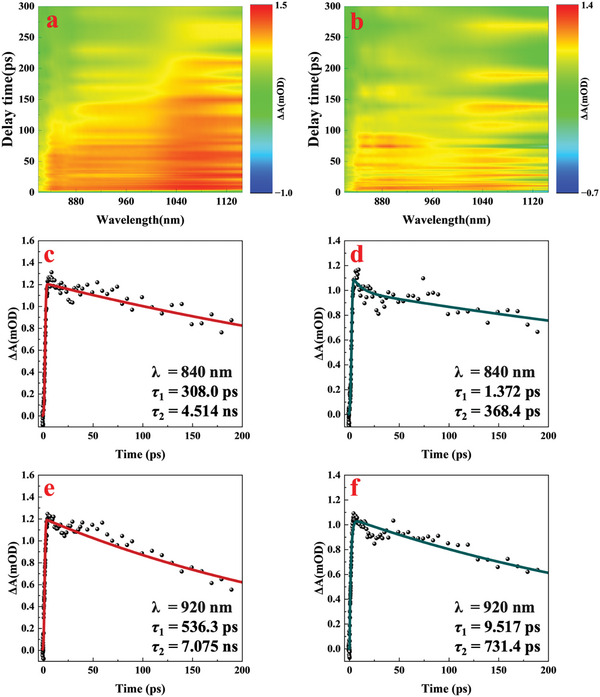
2D decay spectrum of transient absorption ΔA(λ, t) in the spectral range of 820–1145 nm of a) pristine SnSe nanoflakes and b) Cu‐functionalized SnSe nanoflakes. Extracted TAS as functions of pump‐probe delay time with fitting curves of c) pristine SnSe nanoflakes d) Cu‐functionalized SnSe nanoflakes at a wavelength of 840 nm, e) pristine SnSe nanoflakes and f) Cu‐functionalized SnSe nanoflakes at a wavelength of 920 nm.

Femtosecond transient absorption spectroscopy (TAS) was utilized to investigate changes in carrier dynamics after copper functionalization of SnSe nanoflakes. A 400 nm (3.1 eV) pulsed laser served to excite electrons from the valence to the conduction band for both samples. The transient absorption ΔA(λ, t) for pristine and Cu‐functionalized SnSe nanoflakes was observed in the spectral range of 820–1145 nm, as shown in **Figure** **4**a,b. The initial negative ΔA signal near zero delay time post‐pump excitation indicates ground‐state bleaching. Subsequently, a positive ΔA signal, indicating excited‐state absorption, was observed, followed by a gradual decrease in ΔA, which is consistent with the phenomenon in other studies.^[^ ^19^, ^49^ ^]^ The slow recovery time of SnSe could be attributed to the persistence of carriers in defect states that do not readily contribute to recombination.^[^ ^50^ ^]^

To investigate the carrier decay dynamics in pristine SnSe and Cu‐functionalized SnSe nanoflakes, bi‐exponential fitting was applied to the ΔA dynamics curves at 840 nm (Figure 4c,e) and 920 nm (Figure 4d,f), with the following equation:

(1) $$\Delta A\left(t,\omega \right)=a\left(\omega \right)\cdot e^{-t/\tau_{1}}+b\left(\omega \right)\cdot e^{-t/\tau_{2}}+c\left(\omega \right)$$

where *a* and *b* are constants, and *t* represents the delay time between the pump and probe pulse. The relaxation lifetime is characterized by two‐time constants: fast time constant, *τ*₁, attributed to the cooling of hot electrons in the conduction band, and slow time constant *τ*₂, reflecting the carrier interband recombination process. For pristine SnSe nanoflakes illuminated at 840 nm, the relaxation lifetimes were *τ*₁ = 308.0 ps and *τ*₂ = 4.514 ns, compared to *τ*₁ = 1.372 ps and *τ*₂ = 368.4 ps for Cu‐functionalized SnSe nanoflakes at the same wavelength, and at 920 nm, pristine SnSe nanoflakes showed *τ*₁ = 536.3 ps and *τ*₂ = 7.075 ns, versus *τ*₁ = 9.517 ps and *τ*₂ = 731.4 ps for Cu‐functionalized SnSe nanoflakes, this trend was consistent across various wavelengths. The acceleration of fast and slow relaxation times in Cu‐functionalized SnSe nanoflakes, relative to pristine SnSe, arises from multiple factors. Initially, Cu incorporation reduces defect density, thereby diminishing the number of recombination centers and enhancing carrier recombination speed, and higher carrier concentration can enhance the Auger recombination process.^[^ ^51^ ^]^ Furthermore, increased carrier concentration and mobility within Cu‐functionalized SnSe nanoflakes contribute to faster carrier relaxation. Additionally, strain induced by Cu functionalization modifies the band structure, potentially altering carrier relaxation pathways and shortening relaxation durations.

Such significant advancements in carrier dynamics, evidenced by reduced relaxation times in Cu‐functionalized SnSe nanoflakes, underscore copper functionalization's role in augmenting the efficacy of SnSe‐based optoelectronic devices.

### 3.4 Nonlinear Optical Properties Characteristic

**Figure 5:**
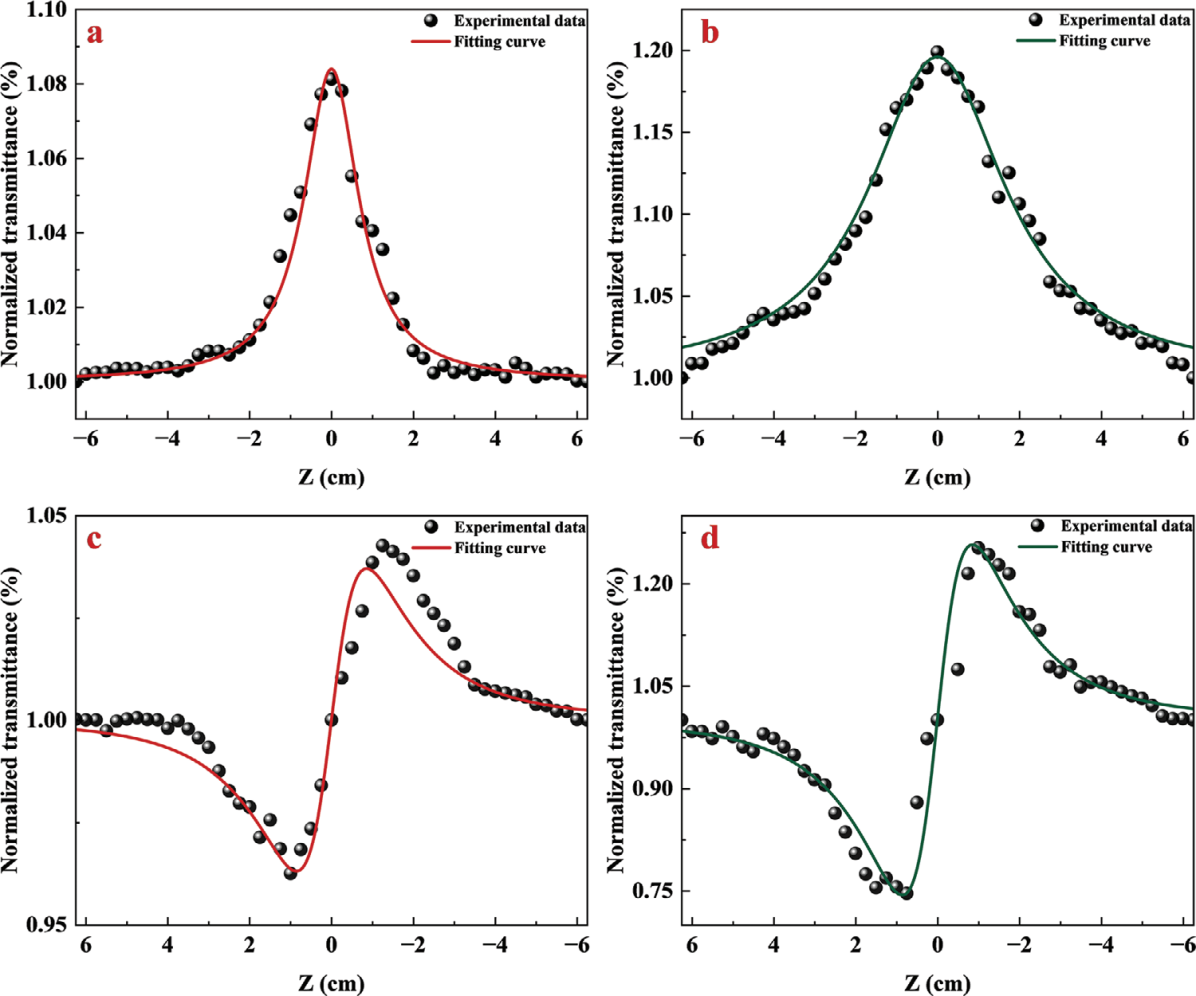
OA *Z*‐scan measurement of a) pristine SnSe nanoflakes and b) Cu‐functionalized SnSe nanoflakes; CA *Z*‐scan measurement of c) pristine SnSe nanoflakes and d) Cu‐functionalized SnSe nanoflakes.

Open‐aperture (OA) and closed‐aperture (CA) *Z*‐scan tests in the 1550 nm are conducted to study the nonlinear optical properties of SnSe after Cu‐functionalization. **Figure** **5**a,b are the OA *Z*‐scan curves of pristine SnSe nanoflakes and Cu‐functionalized SnSe nanoflakes, the nonlinear absorption coefficient β_*eff*_ can be obtained by fitting with the following formula^[^ ^52^ ^]^:

(2) $$\alpha =\alpha_{0}+\beta_{eff}I$$

(3) $$T=\sum_{m=0}^{\infty }\frac{{\left[-q_{0}\left(z,0\right)\right]}^{m}}{{\left(m+1\right)}^{1.5}},\left(m\in N\right),q_{0}\left(z,0\right)=\frac{\beta_{eff}L_{eff}I}{\left(1+z^{2}/z_{0}^{2}\right)}$$

where *I* is the peak intensity at the focus point, *T* and *z* are normalized transmittances, and the relative distance of the sample along the z‐axis. *z*_0_ is the Rayleigh length, and $L_{eff}=\left(1-\frac{e^{L\alpha_{0}}}{\alpha_{0}}\right)$ is the effective length of the testing samples. The fitting results show that the β_*eff*_ of pristine SnSe nanoflakes and Cu‐functionalized SnSe nanoflakes were −0.81 and −2.02 cm GW^−1^, respectively.

Figure 5c,d are the CA *Z*‐scan curves of pristine SnSe and Cu‐functionalized SnSe, which can be fitted using the following formula^[^ ^52^ ^]^:

(4) $$T=1+\frac{4(Z/Z_{0})\Delta \phi }{\left({\left(Z/Z_{0}\right)}^{2}+9\right)\left({\left(Z/Z_{0}\right)}^{2}+1\right)}$$

where Δϕ ≅ *n*_2_ *IkL_eff_* represents the nonlinear phase shifts, and k is the vacuum propagation constant. The nonlinear refractive indices *n*_2_ of pristine SnSe nanoflakes and Cu‐functionalized SnSe nanoflakes were 1.3 × 10^−2^ and 8.7 × 10^−2^ cm^2^ GW^−1^, respectively.

The observed increase in both β_*eff*_ and *n*_2_ values for Cu‐functionalized SnSe suggest a substantial enhancement in nonlinear optical properties. The increase in nonlinear optical responses following Cu‐functionalization not only supports the effectiveness of our Cu‐functionalization strategy but also suggests that Cu‐functionalized SnSe nanoflakes could be considered potential candidates for advanced ultrafast photonic applications.

The SnSe and Cu‐functionalized SnSe nanoflakes SA were fabricated using the optical deposition method. The nanoflakes were deposited on patch cables with an insertion loss of ˂0.05 dB, which was essentially identical. The patch cable was connected to a 974 nm laser diode with an output power of 150 mW and the patch cable was immersed in the nanoflake solution, the ethanol in the solution on the fiber core surface evaporated due to the photothermal effect, and the nanoflakes in the solution would deposit on the fiber core surface simultaneously. After a 2‐h deposition, the patch cable was sealed and dried for 36 h. Sandwich structures are used instead of tapered fibers or side polished fiber (SPF) for the preparation of SAs because of the large insertion loss differences between the different devices of the latter and the tapered fiber and SPF exhibit a noticeable polarization‐dependent effect,^[^ ^53^, ^54^ ^]^ these could introduce impact when comparing the performance differences of these two SAs.

**Figure 6:**
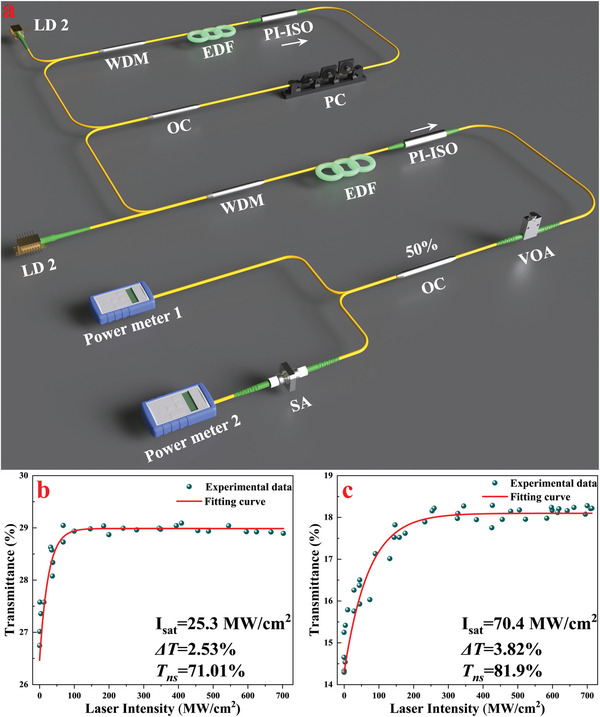
a) Diagram of dual‐balanced detection fiber system and the nonlinear transmittance of b) SnSe and c) Cu‐functionalized SnSe SA.

The nonlinear optical properties of these SAs were investigated using a homemade dual‐balance detection fiber system, the diagram of which is shown in **Figure** **6**a. A homemade nonlinear polarization rotation EDFL was utilized as a pulsed signal source at a wavelength of 1.5 µm. The signal was then amplified by an erbium‐doped fiber amplifier to produce a pulsed laser with a pulse width of ≈830 fs and a repetition frequency of 14.1 MHz. The laser beam passes through a variable optical attenuator to regulate the intensity of light entering the SA. An output coupler (OC) splits the light into two beams with equal intensity. One beam serves as a reference light, while the other passes through the SA. The SA's important parameters are obtained by plotting an equation‐adjusted curve based on a series of collected transmission data. The recorded data were fitted with the following equation:^[^ ^55^ ^]^

(5) $$T=1-\Delta T/\left(1+I/I_{\mathrm{sat}}\right)-\alpha_{0}$$

where *T* is the transmission, Δ*T* is the modulation depth, *I* is the incident intensity, *I*_sat_ is the saturation intensity, and α_0_is the unsaturable loss.

As depicted in Figure 6b,c, the nonlinear transmission of the SnSe and Cu‐functionalized SnSe sandwich structure SA increases with the excitation intensity, exhibiting the typical saturable absorption property. By fitting the nonlinear transmission with Equation (1), the modulation depth Δ*T* of SnSe improved to 3.82% from 2.53% to 3.82% after Cu‐functionalization. Additionally, the saturation strength of Cu‐functionalized SnSe increased remarkably due to the improved electric conductivity and structural integrity. This allows the material to withstand higher optical intensities without degrading, thereby supporting greater saturation strength. Furthermore, Cu‐functionalization significantly alters the electronic structure of SnSe. The introduction of Cu atoms creates new electronic states near the Fermi level, resulting in a more favorable absorption cross‐section for the material. This modification enhances the material's optical properties and contributes to its increased saturation strength by facilitating more efficient light‐matter interactions within the fiber laser's operational regime.

## 4 Application for Ultrafast Photonics

### 4.1 The Conventional Soliton (CS) Mode‐Locking Operation

**Figure 7:**
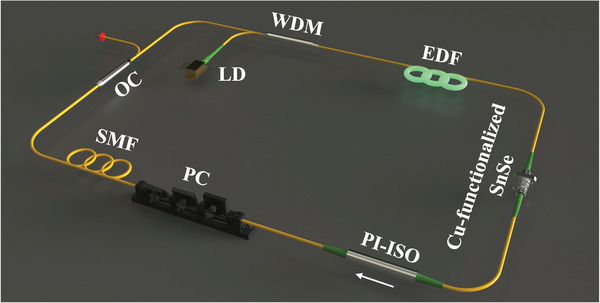
Schematic diagram of the EDFL.

The EDFL with the structure shown in **Figure** **7** has been designed to investigate the ultrafast photonics application of SAs. The fiber laser ring cavity includes a laser diode (LC96Z580‐74, II‐VI Inc.) with a central wavelength of 974 nm was utilized as the excitation source, and a 1.5‐meter high‐absorption erbium‐doped gain fiber (I‐25(980/125), MFD = 5.3–6.3 µm@1550 nm, NA = 0.23–0.26, Fibercore Inc.) with the group velocity dispersion (GVD) of 40 ps^2^ km^−1^ at 1550 nm, a 980/1550 nm wavelength division multiplexer is used to couple the pump source beam into the laser cavity and an OC is used to filter 10% of the laser out, a polarization‐independent isolator is used to contain the reverse light propagation, a polarization controller (PC) is used to control the intra‐cavity polarization state and the sandwich structure SA is used to generate polarization‐related losses. All optical components were connected via single‐mode fiber (SMF‐28e, Corning Inc.) with a GVD of −22.3 ps^2^ km^−1^. The total laser cavity length was measured to be 26.3 m, leading to a net dispersion of −0.49 ps^2^, which is within the anomalous dispersion regime.

**Figure 8:**
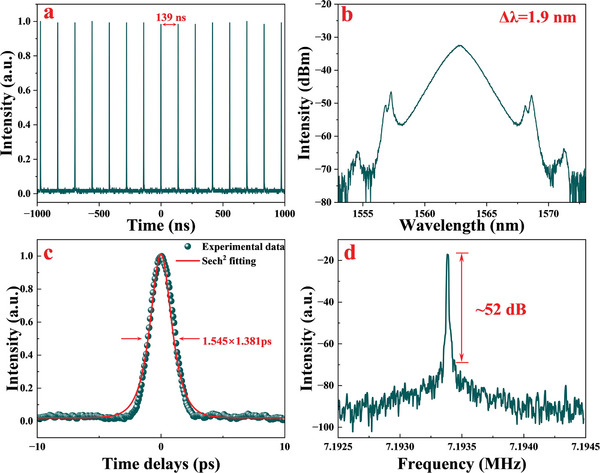
a) The temporal trains, b) corresponding optical spectrum, c) autocorrelation trace, and d) RF spectrum of the CS mode‐locking operation with SnSe‐based SA.

By optimizing the polarization state at a pump power of 120 mW, we were able to achieve CS mode‐locking operation with SnSe‐based SA integrated into the EDFL. Increasing the pump power to 220 mW, CS mode‐locking operated stably and the corresponding experimental record is shown in **Figure** **8**. The oscilloscope trace of the pulse train is shown in Figure 8a, the temporal pulse trains were stable and clutter‐free. Figure 8b shows the optical spectrum of stable CS mode‐locking operation, no sharp peak of the continuous wave component was observed in the spectrum, the central wavelength of the spectrum was 1562.9 nm, the 3 dB bandwidth was ≈1.9 nm, and the sharp and symmetrical Kelly sidebands were observable. The autocorrelation trace is shown in Figure 8c, and the temporal width (full width at half maximum, FWHM) of the pulse was measured as 1.381 ps after a sech^2^ profile fitting. The estimated time‐bandwidth‐product (TBP) value is ≈0.322. The radio‐frequency (RF) spectrum is shown in Figure 8d, the repetition frequency was 7.19 MHz, which corresponds to the temporal pulse period 139 ns, and the signal‐to‐noise ratio (SNR) was 52.4 dB, indicating stable operation of the cavity.

**Figure 9:**
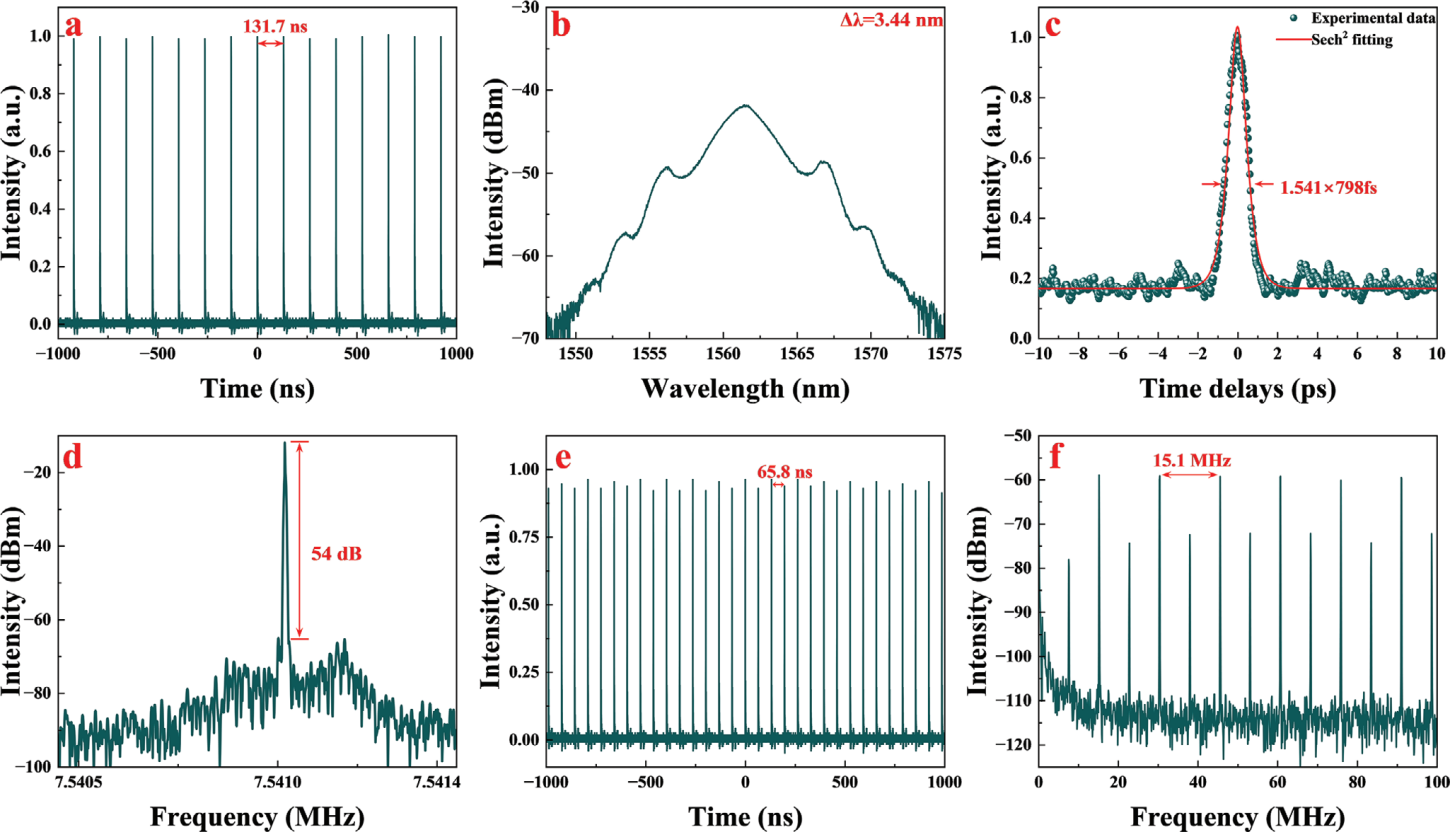
a) The temporal trains, b) corresponding optical spectrum, c) autocorrelation trace, and d) RF spectrum of the CS mode‐locking operation with Cu‐functionalized SnSe‐based SA. e) The temporal trains, b) corresponding RF spectrum with 100 MHz span of second harmonic CS mode‐locking operation.

The Cu‐functionalized SnSe‐based SA replaced the SnSe‐based SA in the same EDFL. By adjusting the polarization controller at a pump power of 50 mW, a Q‐switched operation appeared. Increasing the pump power to 80 mW, CS mode‐locking operation appeared. Further increasing the pump power to 185 mW, the EDFL operated stably in the CS mode‐locking state. The oscilloscope trace of the pulse train is shown in **Figure** **9**a, the temporal pulse period between pulses was 131.7 ns, which agreed with our cavity length, and the slight decrease in period can be attributed to the shortened length of the SMF during the replacement of the SA, which has a negligible impact on the laser operating performance. Figure 9b shows the spectrum of stable CS mode‐locking operation, the central wavelength of the spectrum was 1561.4 nm, and the 3 dB bandwidth was ≈3.44 nm. The Kelly sidebands were symmetrically distributed on both sides. Compared to the performance of the SnSe‐based SA integrated cavity, the center wavelength slightly blue‐shifted by 1.5 nm, and the 3 dB bandwidth increased by 1.54 nm. The autocorrelation trace is shown in Figure 9c, the temporal width (FWHM) was measured as 798 fs after fitting a sech^2^ profile. The estimated TBP value is ≈0.3376, which was higher than the Fourier transform limitation (0.315). The RF spectrum is shown in Figure 9d, the repetition frequency was 7.59 MHz, corresponding to a temporal pulse period of 131.7 ns. The SNR was 52.2 dB, indicating the generation of a highly stable laser pulse signal. Additionally, increasing the pump power to 400 mW, a second harmonic mode‐locking operation will appear, and the oscilloscope trace of the pulse train is shown in Figure 9e, with a temporal pulse period of 65.8 ns, corresponding to 15.18 MHz, which is twice the fundamental frequency. The RF spectrum with a 100 MHz span is shown in Figure 9f. The even multiples of the fundamental frequency yield a higher SNR compared to the odd multiples, indicating the stable operation of the second harmonic mode‐locked state.

**Table** **1** provides a detailed comparison of the performance of various 2D material‐based SAs in mode‐locked laser outputs. Cu‐functionalized SnSe displays a significant improvement in most aspects of performance compared to pristine SnSe. Additionally, Cu‐functionalized SnSe demonstrates superior performance in achieving a shorter pulse width and broader 3dB bandwidth when compared to those sandwich structure SAs of other 2D materials, making it a leading candidate for future developments in ultrafast photonic devices. The study shows that Cu‐functionalization has been successfully applied to enhance the nonlinear optical properties of SnSe, and these insights contribute to the broader application of 2D materials in photonic technologies.

**Table 1:** Performance of mode‐locked laser integrated with various 2D material‐based SAs.

Material	SA Structure	λ [nm]	3 dB bandwidth [nm]	Pulse width [ps]	SNR [dB]	Reference
Graphene	Fiber ferrule	1571.96	0.75	2600	51	[56]
Graphene	SPF	1559	3.44	0.91	–	[56]
BP	Fiber ferrule	1571.45	2.925	0.946	70	[6]
TiS_2_	Fiber ferrule	1569.5	2.63	1.04	66	[11]
Cr_2_Sn_2_Te_6_	Fiber ferrule	1530.1	3.55	1.26	60	[57]
Ta_2_NiS_5_	Fiber ferrule	1569	4.89	1.45	67	[58]
Bi_2_Te_3_–Sb_2_Te_3_	Fiber ferrule	1557.02	5.82	1.62	60.29	[59]
Cu/In2Se3	Fiber ferrule	1562.6	0.8	3.59	76.99	[60]
SnSe	Fiber ferrule	1562.9	1.54	1.381	52.4	This work
Cu‐functionalized SnSe	Fiber ferrule	1561.4	3.44	0.798	52.2	This work

### 4.2 The Bound‐State (BS) Mode‐Locking Operation

**Figure 10:**
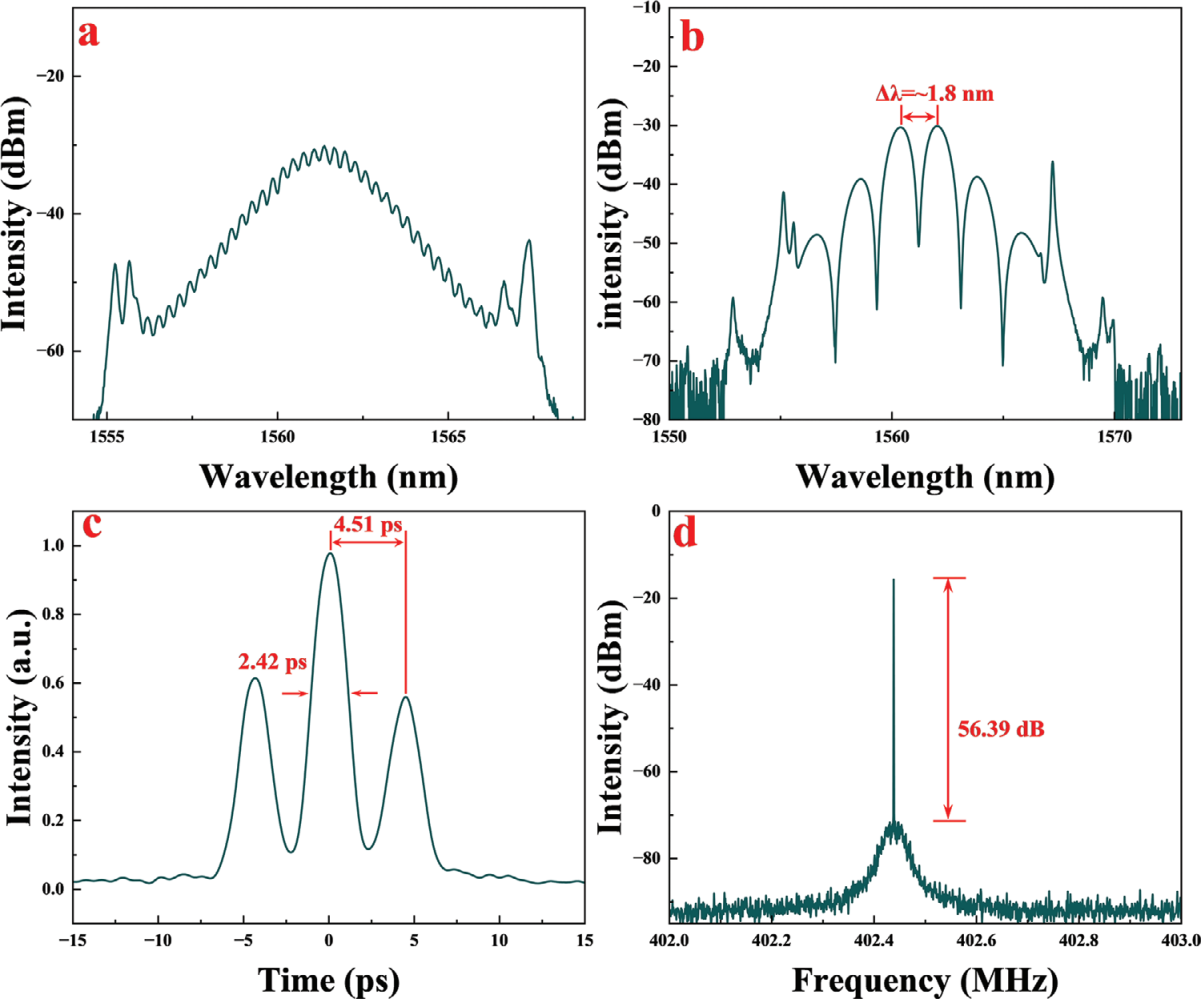
a) The loosened BS spectrum, b) The BS spectrum, c) the corresponding autocorrelation trace, and d) the RF spectrum of the 53rd HML.

Through further adjustments to the polarization state and pump power, the optical spectrum evolved from the CS state to the stable BS state, as shown in **Figure** **10**a,b. The optical spectrum in Figure 10a displayed small soliton molecules, which gradually evolved into a stable BS state in Figure 10b.^[^ ^61^ ^]^

**Figure 11:**
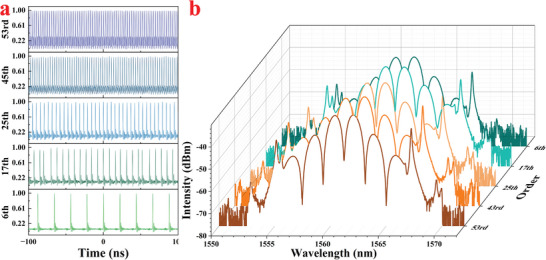
a) The optical spectrum and b) pulse train traces of different orders of HML.

The autocorrelation trace of the BS mode‐locking operation is displayed in Figure 10c. Three neighboring peaks are observed due to the merging of two solitons of identical strength into a single BS pulse. The FWHM of the primary peak measures 2.42 ps, while the time interval between the solitons is 4.51 ps, correctly corresponding to the modulation distance of 1.8 nm of the corresponding spectrum, which is characteristic of BS mode‐locking. Figure 10d shows the RF spectrum of the laser output, with a stable 53rd harmonic mode‐locking operation indicated by the ≈ 56.4 dB SNR peak centered at 402.44 MHz. By fixing the polarization controller and adjusting the pump power from 50 to 250 mW, BS mode‐locking operation increased from the 6th harmonics to the maximum 53rd order. The pulse train traces and optical spectrums of various harmonic order numbers are shown in **Figure** **11**a,b, respectively. The corresponding temporal periods of pulses are 21.95, 7.75, 5.27, 2.927, and 2.485 ns, corresponding to the 6th, 17th, 25th, 45th, and 53rd harmonics, and the spectrums of different order harmonics maintain a similar shape.

## 5 Conclusion

In summary, 2D Cu‐functionalized SnSe nanoflakes were synthesized via a solvothermal method augmented with UALPE. This approach addresses the limitations of SnSe, such as low conductivity, wide bandgap, and insufficient NIR absorption, while enhancing its optical properties for ultrafast photonics applications. By conducting rigorous DFT calculations and experimental characterization, it has been demonstrated that 2D Cu‐functionalized SnSe nanoflakes display significantly enhanced nonlinear optical effects in laser interaction scenarios compared to their pristine SnSe counterparts. The integration of these nanoflakes‐based SAs into an EDFL system resulted in a significant reduction in pulse duration to 789 femtoseconds and an expansion of the 3 dB spectral bandwidth to 3.44 nm. These advancements highlight the critical role of copper functionalization in enhancing the effectiveness of SnSe for next‐generation ultrafast photonic devices. This research not only advances the field by introducing a novel material that outperforms existing solutions but also provides a promising direction for enhancing the properties of wide bandgap 2D materials through targeted functionalization.

## 6 Experimental Section

### Materials Characterization

The samples' surface morphology, microstructure, and optical properties were analyzed using TEM, XPS, UV/VIS/NIR Spectroscopy, and XRD. TEM images, HRTEM images, and selected area electron diffraction maps were observed using a TEM system (FEI Talos F200X G2). Elemental mapping was observed using a STEM system with EDS (Super‐X). XPS measurements were carried out on an X‐ray photoelectron spectrometer (Thermo Fisher Nexsa G2). XRD measurements were performed on an X‐ray diffractometer (Ultima IV).

### Laser Performance

The digital oscilloscope (DSOX6004A, 4 GHz bandwidth, and 20 GSa S^−1^ sampling rate, Keysight Technologies Inc.) with a High‐speed InGaAs PIN photodetector module (PD03, 3 GHz bandwidth) was used to record the pulse trace of the mode‐locking laser. The spectral data was recorded using a telecom optical spectrum analyzer (AQ6370D, YOKOGAWA Inc.) with a resolution of 0.02 nm. The RF spectrum was measured using an RF signal analyzer (FPC1500, ROHDE & SCHWARZ Inc. Germany), while the pulse profile was measured using a second‐harmonic‐generation autocorrelator (FR‐103XL, Femtochrome Research Inc.).

## Conflict of Interest

The authors declare no conflict of interest.

## Data Availability

The data that support the findings of this study are available from the corresponding author upon reasonable request.
